# Yellow barley *xan-m* mutants are deficient in the motor unit SECA1 of the SEC1 translocase system

**DOI:** 10.1007/s00425-025-04654-9

**Published:** 2025-02-26

**Authors:** David Stuart, Anastasiia Ivanova, Shakhira Zakhrabekova, Mats Hansson

**Affiliations:** 1 Department of Biology, Lund University, Lund, Sweden; 2 Present Address: Department of Biology, Norwegian University of Science and Technology, Trondheim, Norway

**Keywords:** Chlorophyll, Chloroplast, *Hordeum vulgare*, Secretory pathway, Xantha

## Abstract

Chloroplast protein transport depends on the SEC1 translocase. Barley *xan-m* mutants, deficient in SECA1, lack chlorophyll and die as seedlings. Their yellow phenotype indicates that carotenoid chemistry is less SEC1-dependent.

Chloroplast proteins encoded by genes located in the cell nucleus need to be transported across up to three chloroplast membranes to find its correct location. SEC1 is one of the major translocase systems. In plants, SEC1 consists of three proteins (SECA1, SECY1 and SECE1) and transports substrate proteins over the thylakoid membrane. SECA1 is an ATPase that delivers the substrate protein to the SECY1–SECE1 channel. In the present study, we analyzed five allelic barley *xan-m* mutants, which had been isolated between 1925 and 1957. The mutants belong to a larger collection of barley mutants deficient in chlorophyll biosynthesis and chloroplast development. Mutations in the *xan-m* gene are recessive and result in a yellow phenotype due to lack of chlorophyll and presence of carotenoids. Mutant seedlings die after approximately 10 days. We identified the defective gene in the *xan-m* mutants by a variant of bulk segregant analysis. The gene *xan-m* is an orthologue of *SECA1* in Arabidopsis. Previously, only genes related to chlorophyll biosynthesis have been identified in the collection of barley *xan* mutants. The yellow phenotype of the mutants demonstrates that proteins responsible for carotenoid biosynthesis and storage are not or less dependent on an intact SEC1 translocase.

**Supplementary Information:**

The online version contains supplementary material available at 10.1007/s00425-025-04654-9.

## Introduction

The chloroplast is the site of photosynthesis. It is generally believed that the chloroplast has evolved from an endosymbiotic cyanobacterium. The presence of a retained genome in the chloroplast is in alignment with this hypothesis. However, during the evolutionary process, most of the chloroplastic genes have been relocated to the cell nucleus. Therefore, the chloroplast is highly dependent on import of proteins from the host cell. These proteins are encoded by nuclear genes and the proteins must be transported over several membranes to reach their destinations in the chloroplast. The first two membranes in chloroplasts are the outer and the inner envelope membranes. The dominating transporters of proteins over these membranes are the Toc (Translocon at the outer chloroplast envelope) and Tic (Translocon at the inner chloroplast envelope) translocases. So-called transit peptides in the N-terminal end of the substrate proteins direct them to the Toc/Tic translocases. The transit peptide is removed in the stroma after completion of the import. Some proteins have additional targeting signals since they should be transported back to the inner envelope membrane or further transported to the thylakoids where for example the entire photosynthetic machinery is located. The responsible translocases for this internal chloroplast protein trafficking and sorting are Sec1 (Secretory pathway 1), Sec2 (Secretory pathway 2), SRP (Signal Recognition Particle) and Tat (Twin arginine translocation) (Celedon and Cline 2013). Each translocase is composed of several proteins encoded by different genes. Interestingly, these transporters are similar to translocases of prokaryotes.

The Sec1 translocase includes an ATPase, SecA1, and two other proteins named SecY1 and SecE1. The two later form a channel through the thylakoid membrane (Schuenemann et al. 1999; Fernandez 2018). SecY1 has ten transmembrane domains and forms the major protein-conducting channel. SecE1 has a single transmembrane domain and contributes to the stability of the complex. SecA1 goes through cycles of ATP binding and hydrolysis, which drives the stepwise translocation of unfolded substrate peptides through the SecY1–SecE1 channel to the destination in the thylakoid. The Sec1 translocase is particularly important for soluble lumenal proteins or thylakoid membrane proteins with large lumenal domains (Fernandez 2018). Sec2 has a similar architecture as Sec1 and consists of three components (SecA2, SecY2 and SecE2), which are localized to the inner envelope membrane. SecE2 was the last to be identified and is less similar to its Sec1 counterpart than SecA2 and SecY2 (Li et al. 2015; Anderson et al. 2019). The Tat translocase is named after the twin arginine residues in the targeting signal sequence of Tat substrate polypeptides. The Tat complex comprises three membrane proteins: TatA, TatB and TatC named Tha4, Hcf106 and TatC, respectively, in Arabidopsis. In the translocation process, the substrate protein first binds a TatB–TatC receptor complex through a close contact between TatC and the double arginine motif of the substrate signal peptide. Second, TatA associates with the TatB–TatC receptor complex in the presence of a transmembrane electrochemical gradient and the translocation is completed. The Tat machinery is typically used for translocation of lumenal proteins that are folded or have bound co-factors (Cline and Theg 2007). Based on predictions from genomic data, approximately equal amounts of luminal proteins are transported by the Tat and Sec1 systems (Peltier et al. 2002). The SRP system targets for example LHCPs (light-harvesting chlorophyll-binding proteins), which are the most abundant proteins in the thylakoid membrane. The system consists of SRP54, SRP43, FtsY and Alb3. SRP54 binds to a transmembrane domain of LHCP, while SRP43 associates with a hydrophilic 18-residue signature sequence (DeLille et al. 2000; Stengel et al. 2008). SRP54 is a conserved protein also present in prokaryotes, whereas SRP43 is unique to chloroplasts. The SRP54–SRP43–LHCP complex is mediated by FtsY and eventually integrated into the membrane by Alb3. Both SRP54 and FtsY are GTPases and hydrolysis of GTP is required for translocation of LHCP. A paralog of Alb3, named Alb4, has been identified (Benz et al. 2009). Alb4 participates in the SRP-mediated insertion of certain proteins such as cytochrome *f* into the thylakoid membrane (Trösch et al. 2015). It has also been shown that Alb3, but not Alb4, interacts with SecY of the Sec1 translocase (Benz et al. 2009).

Mutations in many of the genes encoding proteins of the above-mentioned translocases cause a white, yellow or pale green phenotype. For example, it was found that loss-of-function mutants of the *SecA1* gene in Arabidopsis or absence of the protein lead to a retrograde signal, imbalanced protein content due to changed gene expression, and eventually prevention of chloroplast development (Liu et al. 2010; Spetea and Aronsson 2012). It was evident that homozygous *SecA1* mutants of Arabidopsis cannot survive after the seedling stage due to photo-oxidative stress (Liu et al. 2010). In addition, mutations in Arabidopsis TatC are severe and cause a nearly white phenotype and lack of internal membranes in the plastids (Motohashi et al. 2001). However, the most severe phenotype is seen in mutants of the Sec2 system which are lethal at the globular embryo stage (Skalitzky et al. 2011). In contrast, mutations in the Arabidopsis *SRP43* and *SRP54* genes are milder and the yellow-green mutants are viable in homozygous form (Amin et al. 1999).

A collection of barley mutants defective in chlorophyll biosynthesis and chloroplast development has been deposited at the Nordic Genetic Resource Center (http://www.nordgen.org). The mutants were induced between 1928 and 1983 by Scandinavian barley breeders and researchers. Initially, irradiation was used but later also chemicals. When optimizing the mutagenic methods, the plant breeders used chlorophyll mutants as indicators because chlorophyll mutants can easily be observed already at the seedling state in the M_2_ generation. The mutants were classified according to the presence or absence of chlorophyll and carotenoids, and according to pigment patterns into yellow Xantha mutants, white Albina mutants, pale green lethal Viridis mutants, pale green viable Chlorina mutants, horizontally striped Tigrina mutants and longitudinally striped Striata mutants (Nielsen 1974; Simpson and von Wettstein 1980; Simpson et al. 1985; Henningsen et al. 1993). Over the years, a handful of the mutants have been characterized and the deficient genes have been described, e.g., genes involved in chlorophyll biosynthesis (Hansson et al. 2002; Olsson et al. 2004; Rzeznicka et al. 2005; Axelsson et al. 2006; Braumann et al. 2014; Stuart et al. 2021) and regulation of chlorophyll biosynthesis (Lee et al. 2003). In the present study, we focused on the five allelic mutants at the *xan-m* locus (Table 1). The mutants show traces of chlorophyll but their overall appearance is that they are yellow (Henningsen et al. 1993). Due to the chlorophyll deficiency, homozygous mutants die approximately 10 days after germination when the energy in the seed is depleted. As a consequence, the *xan-m* mutations need to be kept in heterozygous stocks and each experiment involving plant material needs to start with sorting of yellow homozygous mutants from green heterozygous mutants and green homozygous wild type. Here, we show that the *xan-m* gene encodes SecA1. We further characterize the five available mutant alleles at DNA and protein level. Since it is likely that genes encoding other translocase proteins are represented in the mutant collection, we have made a survey of the chromosomal locations of these genes in the barley genome.

**Table 1:** Description of barley *xan-m* mutants. Information assembled from Henningsen et al. (1993)

Mutant	Mother cultivar	Mutagen	Year of isolation
*xan-m.3*	Gull	Spontaneous	Approx. 1925
*xan-m.48*	Bonus	Spontaneous	1957
*xan-m.53*	Bonus	X-rays	1948
*xan-m.72*	Bonus	Diepoxybutane	Approx. 1956
*xan-m.73*	Bonus	Ethylene oxide	1956

## Materials and methods

### Plant material and growth conditions

Barley cultivars Gull, Bonus and five *xan-m* chlorophyll mutants (*xan-m.3*, *xan-m.48*, *xan-m.53*, *xan-m.72*, *xan-m.73*) were used (Table 1). Plants were generally grown in vermiculite in a growth chamber (6700 Lux, 23 °C, relative humidity 35%, light and dark cycle of 16 h/8 h). For transmission electron microscopy experiments, 30 seeds were planted in plug trays filled with vermiculite and grown in climate chambers set at 16/8 h light/dark, 1100 Lux, 20 °C, and 70% humidity.

### DNA and RNA methods

Oligonucleotides used as primers in PCR reactions are listed in Suppl. Table S1. Extraction of genomic DNA was performed as previously described (Stuart et al. 2021). Total RNA was extracted using the NucleoSpin RNA Plant Kit (Macherey–Nagel, Bethlehem, PA, USA). First-strand cDNA synthesis was done with 2.5 µg of total RNA, using the RevertAid First Strand cDNA Synthesis Kit (Thermo Fisher Scientific, Waltham, MA, USA). The first-strand cDNA was then used as template in a PCR reaction containing 10 µL REDExtract-N-Amp PCR ReadyMix (Sigma-Aldrich, St. Louis, MO, USA), 1 µL of 10 µM forward primer, 1 µL of 10 µM reverse primer, 2 µL first-strand cDNA, 1 µL of Extraction solution (Sigma-Aldrich), 1 µL of Dilution solution (Sigma-Aldrich), 4 µL H_2_O. PCR amplification of long genomic DNA fragments (> 10 kbp) was performed with Q5 High-Fidelity DNA Polymerase (New England Biolabs, Ipswich, MA, USA). The PCR reactions contained 5 µL of 5 × Q5 Reaction Buffer, 0.5 µL of 10 mM dNTP, 5 μL 5 × Q5 High GC Enhancer, 1.25 µL of 10 µM forward primer, 1.25 µL of 10 µM reverse primer, 2.75 µL of genomic DNA, 0.25 µL of Q5 High-Fidelity DNA Polymerase, and 9 µL H_2_O. Samples selected for Sanger DNA sequencing were prepared using the ExoProStar 1-STEP Kit (Sigma-Aldrich). Sanger sequencing was performed by Eurofins using Eurofins Genomics Mix2Seq Kit (http://www.eurofinsgenomics.eu).

### Genetic mapping using an F_2_-mapping population

To create an F_2_-mapping population seeds from a heterozygous *xan-m.53* stock were planted and green plants, being a mixture of heterozygous mutants and homozygous wild types, were crossed to the cultivar Quench. Since the green phenotype of the heterozygous mutants and the wild-type plants are indistinguishable, the progenies had to be sorted to get an F_2_-mapping population based on heterozygous F_1_ plants. To do this, the F_1_ plants were grown to full maturity. One spike with F_2_ seeds from each F_1_ plant was planted. Appearance of yellow and green seedlings, germinating from a spike, demonstrated the heterozygous genotype of the F_1_ plant. The remaining F_2_ seeds of that plant were kept and formed the F_2_-mapping population. Using *xan-m.53* as male parent in the crosses, possible contamination from seeds generated through bad emasculation of the mother plant was avoided. The F_2_-mapping population was grown on 0.25 m^2^ in vermiculated at room temperature for 1 week. The plants were placed on the floor under the lab bench to avoid direct sunlight. Approximately 70 mg from homozygous mutant leaf seedlings were harvested. The leaf material was pooled and genomic DNA was extracted by a modified CTAB protocol as previously described (Stuart et al. 2021). The genomic DNA was prepared for sequencing with a TruSeq PCR free DNA library preparation kit (Illumina Inc., San Diego, CA, USA) and sequenced on a NovaSeq S4 flowcell at SciLifeLab (http://www.scilifelab.se).

### Chlorophyll measurements

Chlorophyll measurements were done on seedling leaves using a Hansatech Instruments CL-01 Chlorophyll Meter (Hansatech Instruments Ltd., King’s Lynn, UK). Each seedling was measured at 5 positions and the average was used as input value of that plant. Significant differences were calculated using a two-sided Student’s *t* test.

### Competent cells’ preparation

Calcium competent *Escherichia coli* BL21 Star™ (DE3) cells were made in order to prepare the cells for the following heat-shock transformation. 1 mL overnight culture of *E. coli* was inoculated in 100 mL Luria–Bertani broth (LB) and the culture grew on a rotary shaker at 37 °C for 2.5 h. The culture was put on ice for 10 min, then transferred to two 50 mL tubes and spun down in a Jouan BR4i centrifuge for 10 min, 4 °C, 3200×*g*. Pellets were resuspended in 50 mL ice cool 0.1 M MgCl_2_. The cells were incubated on ice for 15 min, spun down at the same conditions and the pellet resuspended in ice cool 0.1 M CaCl_2_, incubated on ice for 15 min and spun down (same conditions). The new pellet was resuspended in 5 mL ice cool 0.1 M CaCl_2_. The cells were incubated on ice for 1 h prior to transformation.

### Protein methods

A plasmid named pET15bHvSecA1 for expression of barley *xan-m* was ordered from GenScript (http://www.genscript.com). The synthetic *xan-m* gene, without the N-terminal 41 amino-acid residue chloroplast transit peptide, was codon optimized for expression in *E. coli*. The optimized coding sequence was cloned into pET15b at the *Nde*I and *Xho*I sites, which results in the addition of an N-terminal His-tag fusion to XanM. The recombinant protein was produced in *E. coli* BL21Star(DE3). One liter LB-medium supplemented with ampicillin (100 mg/L) was inoculated to an optical density (OD_600_) of 0.1. The culture was grown at 20 °C on a rotary shaker. At an OD_600_ of 0.4, isopropyl-β-d-thiogalactopyranoside (IPTG) was added to a final concentration of 1 mM. The culture was left to grow overnight. The bacteria were harvested by centrifugation at 10,000×*g* for 20 min at 4 °C and washed once with binding buffer (20 mM imidazole, 0.5 M NaCl, 20 mM Tris–HCl, pH 8.0). Cells from 1 L culture was resuspended in 50 mL Binding buffer with a few crystals of lysozyme and DNase I added. The bacteria were lysed by passage through a French pressure cell two times at 12.4 MPa followed by centrifugation at 3600×*g* for 10 min at 4 °C. XanM was purified from the supernatant using immobilized metal ion affinity chromatography (IMAC) with a 1 mL HisTrap FF column (http://www.cytivalifesciences.com) as previously described (Stuart et al. 2020). The amount of protein in eluted fractions was determined with Bradford reagent (http://www.bio-rad.com) using bovine serum albumin fraction V as a standard. The eluted fraction with most protein was desalted on a NAP 10 column (http://www.cytivalifesciences.com) into 20 mM Tris–HCl pH 8.0. Antibodies were raised against the recombinant XanM protein in rabbit by Agrisera (Vännäs, Sweden). SDS-PAGE and western blot analyses were performed as previously described (Stuart et al. 2020).

### Transmission electron microscopy

Seeds obtained from heterozygous *xan-m.3* and *xan-m.48* mutants were planted and the resulting seedlings were genotyped by PCR using oligonucleotides as primers which could specifically amplify the deletions in *xan-m.3* and *xan-m.48* or the wild-type allele (Suppl. Table S1). One cm^2^ leaf samples were taken from all yellow mutants, green heterozygous *xan-m.3* and *xan-m.48* and Bonus. The transmission electron microscopy was performed at the Microscopy Facility at Department of Biology, Lund University. Fixation, dehydration, embedment, ultratome cutting and staining of the samples were performed as previously described (Henningsen et al. 1993). 50 nm ultra-thin sections were mounted on copper grids, stained with 2% (w/v) uranyl acetate for 30 min and 80 mM lead citrate for 4 min and analyzed with a JEOL JEM 1400 Plus transmission electron microscope, operated at 100 kV.

## Results

### Identification of the *xan-m* gene

**Fig. 1:**
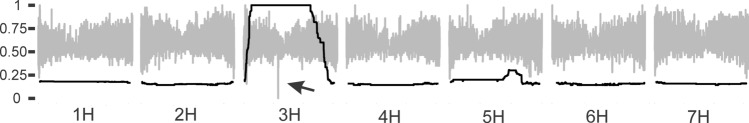
Mapping the *xan-m* gene using an F_2_-mapping population and genome scan for large deletions in *xan-m.53*. The F_2_ population originated from a cross between mutant *xan-m.53* and barley cultivar Quench. Black trace: allele depth difference mapping of the *xan-m.53* mutation to the seven barley chromosomes (1H to 7H). The allele depth difference was calculated as the absolute value of the difference in sequencing depth between the two alleles divided by the sum of the read counts for the two alleles. To denoise the data a running median was calculated from the nearest 15,000 SNPs and plotted along each chromosome. The *xan-m.53* mutation is linked to SNPs in the centromeric region of chromosome 3H with a median allele depth difference of one. Grey trace: the sequencing depth of exons along each chromosome for identification of deletions. Read depth has been min–max normalized to the scale of 0 to 1 to fit the same plot and a running median of the nearest 2000 bp was calculated to denoise the data. Read depth can be seen to drop to zero on chromosome 3H (indicated by an arrow) in the mapping interval for *xan-m.53* due to a large deletion which includes half of the *HvSecA1* gene

In order to identify the *xan-m* gene, an F_2_-mapping population was constructed using the mutant *xan-m.53* and the cultivar Quench. For mapping, approximately, 1000 F_2_ seeds were planted and 242 segregating homozygous mutant seedlings were obtained. Approximately, 70 mg of leaf material was collected from each seedling. The leaf material was pooled and genomic DNA was extracted. From Illumina sequencing, we obtained approximately 448 million raw 150 bp paired-end reads. SNP calling and filtering of data was done as previously described (Stuart et al. 2024). Since *xan-m.53* was induced in the cultivar Bonus, most SNPs should be due to genetic differences between Bonus and Quench. A long stretch of SNPs related to Bonus was found on chromosome 3H. This suggested that *xan-m.53* has a centromeric location on chromosome 3H (Fig. 1). We also explored the genomic sequence data to find mutations in candidate genes that could explain the *xan-m* mutations. Only one gene, HORVU.MOREX.r3.3HG0267340, located in the mapped region, had a point mutation that caused changes of amino-acid residues in the corresponding protein. This gene is orthologous to *SCD1* (stomatal cytokinesis defective 1) in Arabidopsis (Falbel et al. 2003). HORVU.MOREX.r3.3HG0267340 was sequenced also in *xan-m.3*, *xan-m.48*, *xan-m.72* and *xan-m.73* but no mutations were found. Thus, HORVU.MOREX.r3.3HG0267340 is not the *xan-m* gene. We also noted a gap in the mapped region that most likely corresponded to a large deletion in *xan-m.53* (Fig. 1). We, therefore, analyzed the sequencing depth of the mapped region, which revealed the absence of sequenced reads in a region spanning several thousand base pairs. One of the deletion breakpoints was in HORVU.MOREX.r3.3HG0259100. This gene is annotated as Protein translocase subunit SecA1 based on similarities to Arabidopsis.

### Characterization of *xan-m* mutations

**Fig. 2:**
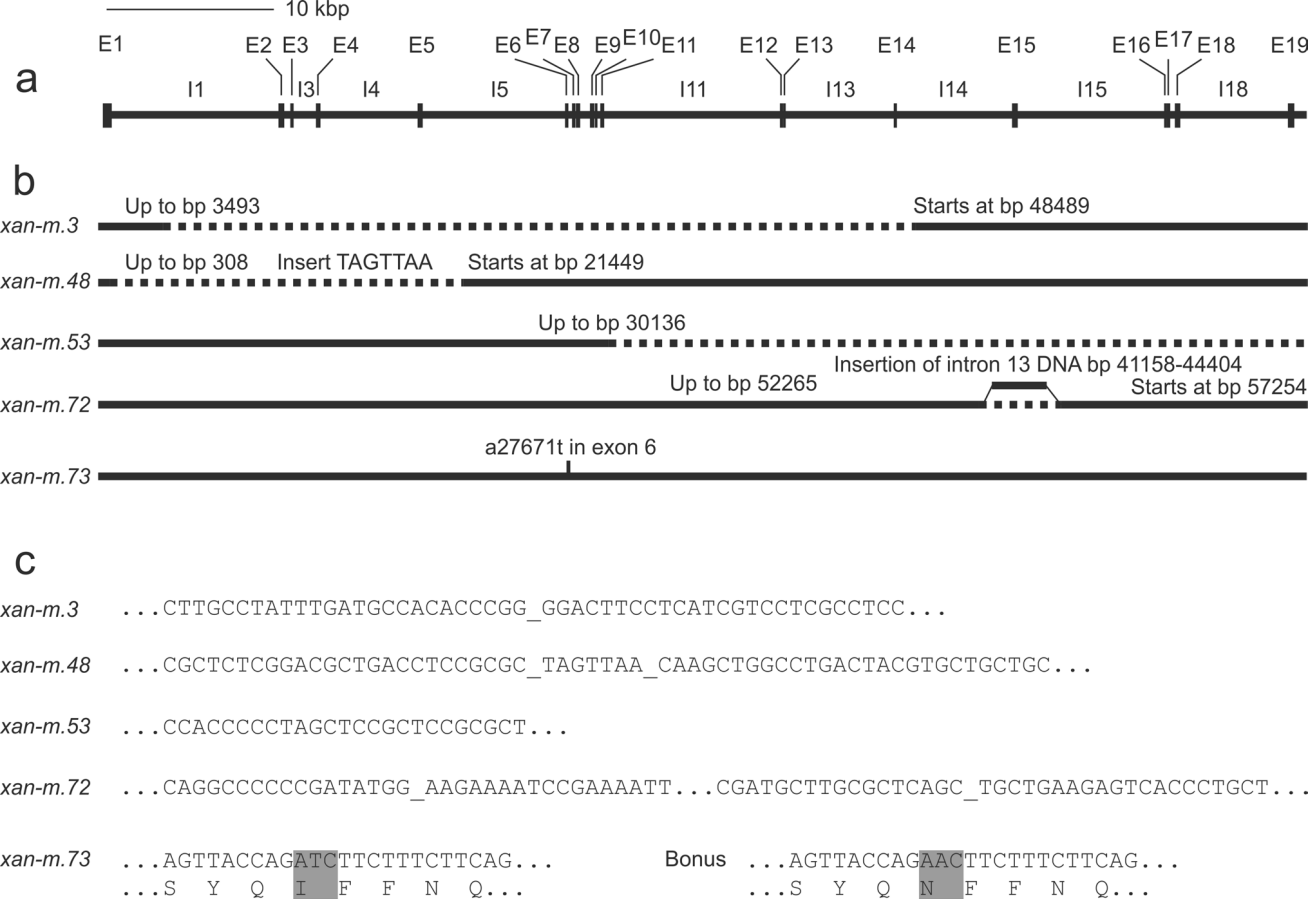
Gene map of barley *xan-m* and location of the identified mutations in *xan-m*.*3*, *xan-m.48*, *xan-m.53*, *xan-m.72* and *xan-m.73*. **a** The gene map starts with the ATG start codon at bp 227,519,289 in Exon 1 (E1). The gene has 19 exons and 18 introns. **b** Graphic representation of the five mutations. Dashed lines indicate the location of deletions. All positions relate to the first bp of the ATG start codon. **c** Detailed view of the five mutations. Deletion breakpoints have been separated by an underline

**Fig. 3:**
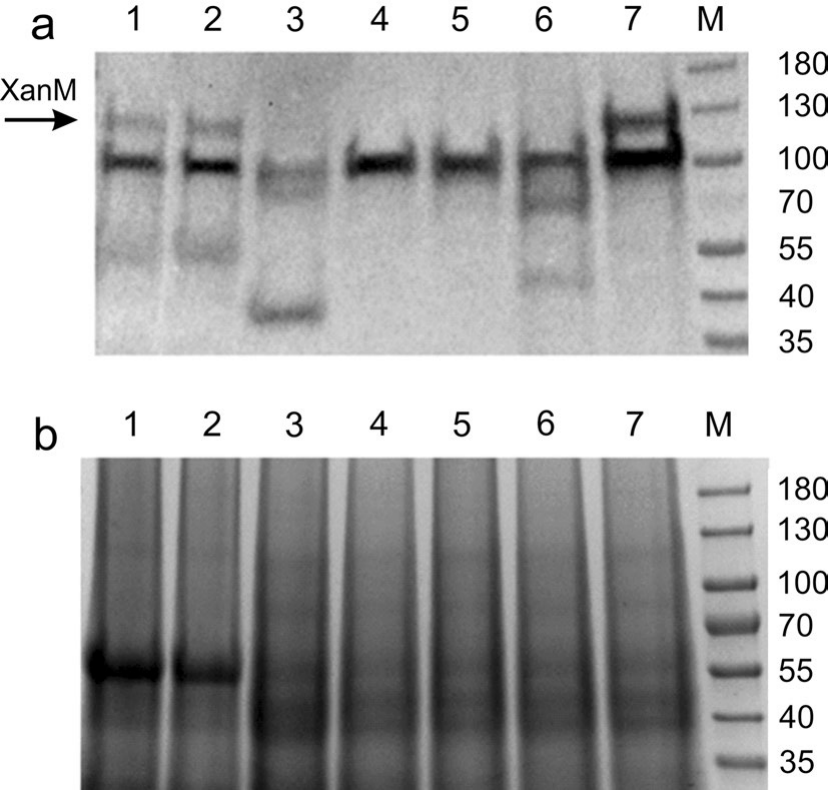
**a** Western-blot analysis of XanM protein in the barley cultivars Bonus, Gull and mutants *xan-m.3*, *xan-m.48*, *xan-m.53*, *xan-m.72* and *xan-m.73*. Total protein was extracted and analyzed. Truncated versions of XanM/SecA are seen in *xan-m.3* and *xan-m.72*. Lane 1, Bonus; lane 2, Gull; lane 3, *xan-m.3*; lane 4, *xan-m.48*; lane 5, *xan-m.53*; lane 6, *xan-m.72*; lane 7, *xan-m.73*; lane M, Thermo Scientific™ PageRuler™ Prestained Protein Ladder. The XanM/SecA protein is marked by an arrow. **b** A replica of the SDS-PAGE gel used for Western-blot analysis stained with Coomassie Brilliant Blue

To verify that HORVU.MOREX.r3.3HG0259100, encoding the transport protein SecA1, is the correct candidate gene, we sequenced the gene from the five available *xan-m* mutants. HORVU.MOREX.r3.3HG0259100 starts with an ATG codon at bp 227,519,289 and ends with a TGA stop codon at bp 227,448,383. It contains 19 exons and 18 introns. The resulting polypeptide consists of 996 amino-acid residues and has a calculated mass of 112 kDa. Due to the large size of the gene, our sequencing strategy was to cover exon DNA sequences by sequencing selected parts of genomic DNA and cDNA. Small, but not large, introns were sequenced. Initial attempts to amplify the exons of the gene with PCR indicated that several of the mutants contained large deletions or chromosomal rearrangements. To reveal the different deletion breakpoints, we performed PCR reactions with various combinations of primers. The deletion in *xan-m.3* is 44,995 bp and starts in Intron 1 and ends in Intron 14 (Fig. 2). The *xan-m.48* deletion is 21,140 bp starting in Exon 1 and ending in Intron 5. It has also a 7 bp insert (TAGTTAA). The *xan-m.53* deletion is over 1.7 Mbp and starts in Intron 11 at bp 30,136. In *xan-m.72*, Intron 14 continues until a breakpoint at bp 52,265, followed by an insertion of DNA from Intron 13 (from bp 41,158 to bp 44,404) with a second breakpoint in Intron 15, starting at bp 57,254. The *xan-m.73* mutant has a point mutation in Exon 6 position 27,671 (A to T). The mutation changes the second nucleotide in a codon (AAC to ATC) and thus replaced an asparagine with an isoleucine. The modified protein in *xan-m.73* could be detected in western blot analysis (Fig. 3). In contrast, no XanM protein could be detected in the total cell extract of *xan-m.48* and *xan-m.53*. Possibly truncated versions of XanM could be detected in *xan-m.3* and *xan-m.72* (Fig. 3). We conclude that the identification of mutations in all five available *xan-m* mutants is a very strong proof that the correct gene had been identified. Thus, HORVU.MOREX.r3.3HG0259100 is *xan-m* (named HORVU3Hr1G041250 and HORVU.MOREX.r2.3HG0215060 in the first and second version of the barley genomes (Colmsee et al. 2015).

### Chlorophyll content and plastid structures in *xan-m* mutants

**Fig. 4:**
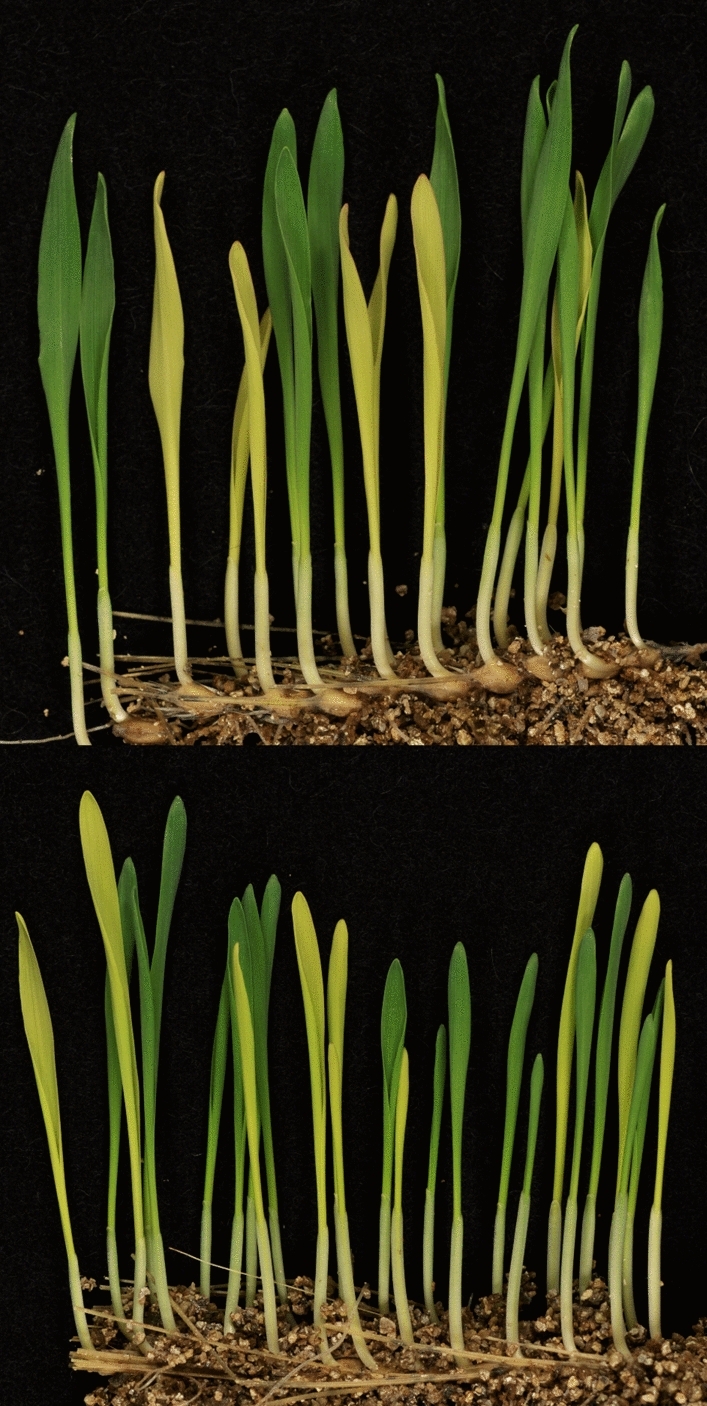
Segregating *xan-m.72* (top) and *xan-m.73* (bottom) mutants from spikes of heterozygous parents. Homozygous mutants are seen as yellow seedlings. Green seedlings are heterozygous mutants or homozygous wild type. The yellow plants die after approximately ten days when the energy reserves in the kernels are depleted

**Fig. 5:**
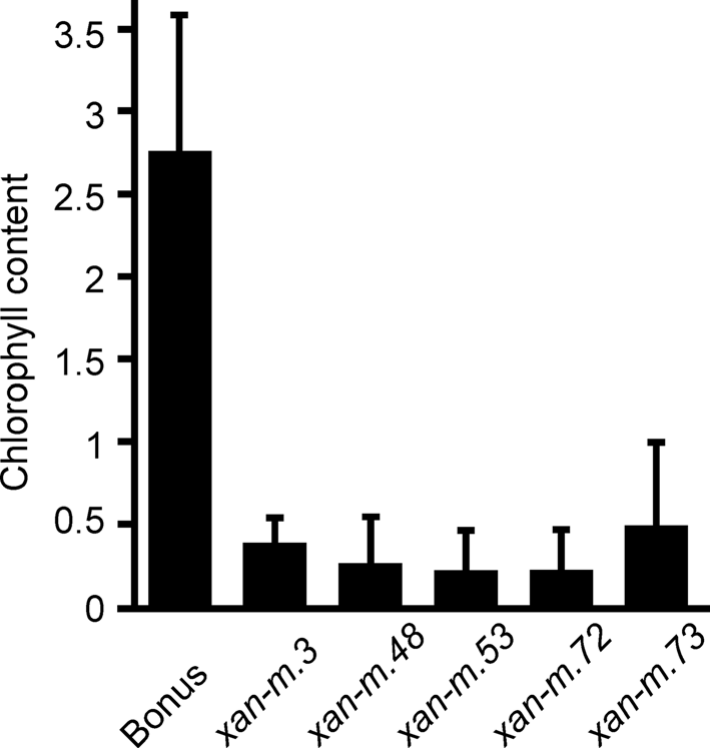
Average chlorophyll content (± standard deviation, *n* = 20) in *xan-m* barley mutant leaves measured with a Hansatech Instruments CL-01 Chlorophyll Meter. Mutant *xan-m.73* had significantly higher level (*P* < 0.001) of relative chlorophyll content than *xan-m.48*, *xan-m.53* and *xan-m.72* but not *xan-m.3*

Chlorophyll biosynthesis and chloroplast development are intimately connected. The recessive mutations in the *xan-m*.*3*, *xan-m*.*48*, *xan-m*.*53*, *xan-m*.*72* and *xan-m*.*73* mutants cause a typical lethal and yellow Xantha phenotype in homozygous leaves (Fig. 4). The plants die at the seedling stage. Earlier descriptions of *xan-m* mutants showed that chlorophyll can be detected also in yellow homozygous mutants but it was not specified which *xan-m* mutant that was analyzed (Henningsen et al. 1993). Especially homozygous mutant leaves of *xan-m.73* show a clear green hue (Fig. 4). To compare the amount of chlorophyll in the different *xan-m* mutants, chlorophyll measurements were done on 17 to 29 intact seedling leaves. It was observed that *xan-m.73* had a significantly higher level of relative chlorophyll content (Fig. 5).

**Fig. 6:**
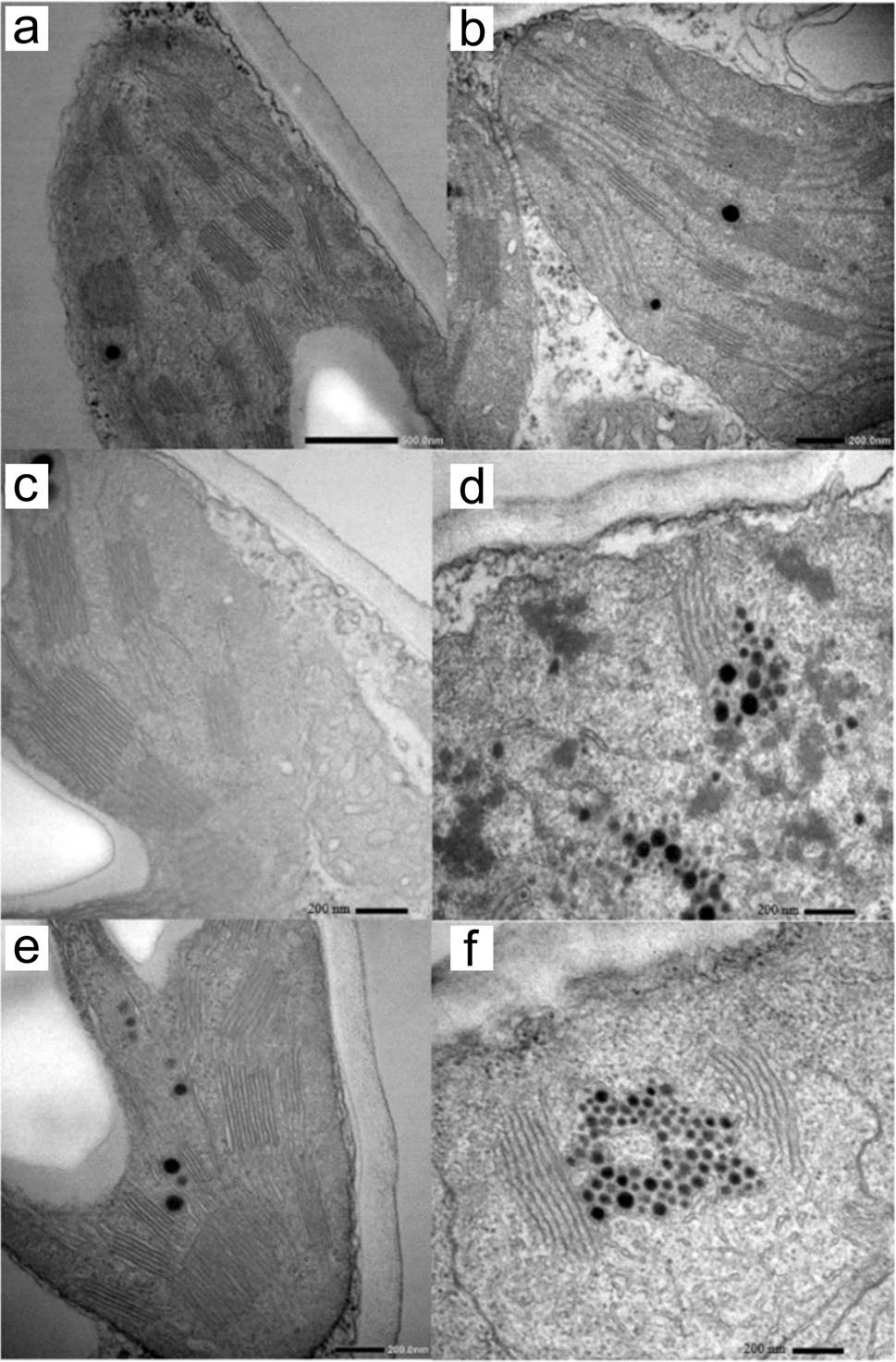
Transmission electron microscopy of barley cultivar Bonus (**a**, **b**), green heterozygous *xan-m.3* (**c**), yellow homozygous mutant *xan-m.3* (**d**), green heterozygous *xan-m.48* (**e**) and yellow homozygous *xan-m.48* (**f**). No major structural differences were found between green tissues of heterozygous mutants and Bonus. Homozygous mutants had undeveloped lamellae systems. Clusters of plastoglobules were common in homozygous mutants

We further performed ultrastructural studies of the lamellar systems of the plastids with transmission electron microscopy. Mutants *xan-m.3* and *xan-m.48* were analyzed. Homozygous mutants showed an undeveloped membrane structure with a few lamellae, which occasionally were arranged in grana-like structures. Clusters of globuli were frequently seen in the mutant plastids (Fig. 6). We also analyzed green leaves in the segregating material. Genotyping of green seedlings was done using PCR with deletion-specific and wild-type specific primers. This allowed us to differentiate between heterozygous mutants and homozygous wild-type seedlings. Both genotypes developed grana stacks of apparent normal size and number (Fig. 6).

### Translocase genes in barley

Sec1, Sec2, SRP and Tat are the translocases responsible for the internal chloroplast protein trafficking and sorting as we know it in plants today. The translocases are composed of several proteins. To identify the barley genes encoding the proteins forming the barley translocases, we searched the literature for genes of Arabidopsis where the functional components have been studied. The polypeptide sequence of a translocase protein was used to search the corresponding barley protein using the IPK Galaxy Blast Suite (https://galaxy-web.ipk-gatersleben.de/). We used the protein sequences deduced from the third version of the barley cultivar Morex genome (Mascher et al. 2021). The search provided the gene name that was used to obtain more information of the gene in the Barlex genome browser (Colmsee et al. 2015). It is likely that each protein component of the three translocases Sec1, Sec2 and Tat is encoded by single genes in the barley genome (Table 2). The SRP translocase seems to be more complex. In this translocase, there are two neighboring and very similar genes encoding SRP54. Two barley orthologues of Arabidopsis ALB3 and ALB4 were found but one of the barley gene products showed highest similarity to both ALB3 and ALB4.

**Table 2:** Identified barley genes encoding putative translocase proteins involved in internal protein trafficking and sorting

Arabidopsis		Identity (%)	Barley	
Name	Accession		Accession	Position (bp)
Sec1 pathway				
SECA1	At4g01800	78	HORVU.MOREX.r3.3HG0259100	227,448,203–227,519,451
SECY1	At2g18710	82	HORVU.MOREX.r3.5HG0467750	346,488,000–346,520,451
SECE1	At4g14870	64	HORVU.MOREX.r3.2HG0159280	405,491,515–405,492,253
Sec2 pathway				
SECA2	At1g21650	74	HORVU.MOREX.r3.4HG0347020	72,479,991–72,494,223
SECY2	At2g31530	75	HORVU.MOREX.r3.1HG0064000	421,020,030–421,032,005
SECE2	At4g38490	60	HORVU.MOREX.r3.5HG0485740	456,402,953–456,405,153
SRP pathway				
ALB3	At2g28800	73	HORVU.MOREX.r3.3HG0230960	24,917,830–24,921,756
ALB3	At2g28800	68	HORVU.MOREX.r3.5HG0532950	576,977,941–576,988,054
ALB4	At1g24490	69	HORVU.MOREX.r3.3HG0230960	24,917,830–24,921,756
ALB4	At1g24490	66	HORVU.MOREX.r3.5HG0532950	576,977,941–576,988,054
SRP43	At2g47450	49	HORVU.MOREX.r3.4HG0409840	587,249,482–587,251,166
SRP54	At5g03940	77	HORVU.MOREX.r3.4HG0351550	108,676,002–108,681,979
SRP54	At5g03940	76	HORVU.MOREX.r3.4HG0351560	108,684,081–108,698,186
FtsY	At2g45770	79	HORVU.MOREX.r3.3HG0323030	602,987,654–602,990,354
Tat pathway				
TatA/Tha4	At5g28750	63	HORVU.MOREX.r3.5HG0509070	523,136,200–523,138,350
TatB/Hcf106	At5g52440	64	HORVU.MOREX.r3.7HG0710620	465,274,403–465,277,384
TatC	At2g01110	83	HORVU.MOREX.r3.3HG0261530	253,297,350–253,330,795

Protein polypeptides obtained from Arabidopsis genes shown in the table were used to screen for similar proteins in barley. The table list high confidence barley genes, which corresponding proteins showed at least 35% identical residues to the Arabidopsis translocase proteins

## Discussion

In the present study, we demonstrate that the barley *xan-m* gene encodes protein SecA1, which is an ATPase that binds the target protein of the Sec1 translocase for import of nuclear encoded chloroplast proteins into the thylakoid lumen and insertion into the membrane. We did this through analyses of five barley *xan-m* mutants, which are characterized by their yellow and lethal phenotype that can be seen in homozygous mutant seedlings. Other *xan* mutants with a similar phenotype have previously been connected to genes encoding chlorophyll biosynthetic enzymes; three subunits of Mg-chelatase (*xan-f*, *-g* and *-h*), Mg-protoporphyrin monomethyl cyclase (*xan-l*) and chlorophyll synthase (*xan-j*) (Hansson et al. 2002; Olsson et al. 2004; Rzeznicka et al. 2005; Axelsson et al. 2006; Braumann et al. 2014). Thus, *xan-m* is the first identified *xan* gene that is not directly involved in chlorophyll biosynthesis.

The yellow color of the *xan-m* mutans demonstrates that they contain carotenoids. This suggests that enzymes involved in carotenoid biosynthesis are not among the many proteins that are transported by the Sec1 translocase, at least not in a SecA1 dependent manner. It should, however, be noted that T-DNA insertion mutants in Arabidopsis *AGY1* (encoding SecA1) had different colors depending on water content in the growing medium; glassy yellow on 0.3% agar and completely albino on 1.0% agar (Liu et al. 2010). An attempt was made to determine the chloroplastic location of carotenoid biosynthetic enzymes (Joyard et al. 2009). Enzymes responsible for the formation of carotenoids, including violaxanthin, are located in the chloroplast envelope membrane. Zeaxanthin epoxidase, catalyzing the formation of violaxanthin, was also found in thylakoid membranes. The dual location of zeaxanthin epoxidase probably reflects its involvement in the xanthophyll cycle, in which also violaxanthin de-epoxidase participates, which is located in the thylakoid membrane.

We also noted that clusters of plastoglobules are abundant in the homozygous *xan-m* mutants (Fig. 4). Plastoglobules are common in etioplasts and decrease as etioplasts are converted into chloroplasts upon illumination. The plastoglobules probably supply lipid building blocks to the membranes during thylakoid formation in greening tissues (Rottet et al. 2015). During this process, they decrease in numbers. In the *xan-m* mutants, we suggest that the plastoglobules remain since the mutants do not form thylakoids. However, accumulation of plastoglobules has also been seen as a response to stress. For example, increased amounts of plastoglobules were found in stressed Arabidopsis *tatC* translocase and *clpR2* protease mutants (Motohashi et al. 2001; Rudella et al. 2006). The plastoglobules have also been found to play a role in adaptation such as remodeling of thylakoid membranes under light stress (Espinoza-Corral et al. 2021). Thus, failure to assemble thylakoids due to *xan-m* mutations might result in accumulation of plastoglobules to store membrane components which cannot be utilized. Transmission electron microscopy photos of plastids of 44 Xantha and 10 Albina mutants have been published (Henningsen et al. 1993). Several of the mutants show accumulation of plastoglobules also in photoperiod cycles of 16 h light/8 h dark. Future analyses of these lines will reveal whether accumulation of plastoglobules in the mutants is correlated with defects in genes encoding chloroplast translocase proteins.

Much of the knowledge concerning transport of proteins over the chloroplast membranes is based on findings in bacterial systems (Wang and Dalbey 2011; Celedon and Cline 2013). The conserved nature of the transportation systems reflects the evolutionary relationship between eubacteria and plant chloroplasts. Most studies in plants have been done in Arabidopsis where all core components of the Sec1, Sec2, SRP and Tat translocases have been studied. Based on genome-wide predictions, approximately half of the proteins allocated to the thylakoid lumen are transported by the Sec1 system and half by the TAT system (Peltier et al. 2002). Mutations in Arabidopsis genes corresponding to these translocases are often yellow, white or pale green (Motohashi et al. 2001; Liu et al. 2010; Skalitzky et al. 2011). Few genetic studies have been published on these translocases in other plants. Three *csy1* mutants deficient in the SecY1 protein of the Sec1 translocase were studied in maize (Roy and Barkan 1998). The three mutant lines had unique *Mu* transposon insertions in different parts of the *csy1* gene resulting in a yellowish phenotype of homozygous mutant seedlings. Thus, studies from Arabidopsis and maize suggest that many genes encoding chloroplast translocases should be possible to identify through analyses of chlorophyll-deficient mutants. We postulate that the many available barley mutants defective in chlorophyll biosynthesis and chloroplast development (Hansson et al. 2024) could be a good source for such studies. We, therefore, explored the accumulated knowledge about Arabidopsis chloroplast translocases to identify the location of the corresponding genes in the barley genome (Table 2), which will provide a foundation for future studies of chloroplast translocases in barley and other grass species.

## Supplementary Information

Below is the link to the electronic supplementary material.

Supplementary file1 (DOCX 21 KB)

## Data Availability

Sequence data from this article can be found in the NCBI Sequence Read Archive (https://www.ncbi.nlm.nih.gov/sra) under accession number PRJNA1203586.

## Abbreviations

Sec: Secretory pathway
SNP: Single-nucleotide polymorphism
SRP: Signal recognition particle
Tat: Twin arginine translocation
