# Supplementary material for: Yellow barley xan-m mutants are deficient in the motor unit SECA1 of the SEC1 translocase system
Source: Planta. 2025 Feb 26;261(4):68. doi: 10.1007/s00425-025-04654-9 (PMC11865152; doi:10.1007/s00425-025-04654-9)
Supplement: Supplementary file 1 — Supplementary file1 (DOCX 21 KB) [file 425_2025_4654_MOESM1_ESM.docx]

**Suppl. Table S1.** List of used oligonucleotides.

| Name | Sequence | Description |
| --- | --- | --- |
| Primers for cDNA |  |  |
| SecA_cDNA_F1 | CTGCACTAGACCACAAGCGA | cDNA Forward primer #1 |
| SecA_cDNA_R1 | CCCAAAAAGCGGGGAACTTG | cDNA Reverse primer #1 |
| SecA_cDNA_F2 | CAGCTGATTGGTGGGATGGT | cDNA Forward primer #2 |
| SecA_cDNA_R2 | TCTGCCTGCCATTACTCGAC | cDNA Reverse primer #2 |
| SecA_cDNA_F3 | AGAACAAGGCTACGCTGACG | cDNA Forward primer #3 |
| SecA_cDNA_R3 | ATATCAGTGCCACGTCCTGC | cDNA Reverse primer #3 |
| SecA_cDNA_F4 | CCGTCCTGTGCTTGTTGGTA | cDNA Forward primer #4 |
| SecA_cDNA_R4 | TGCGGGATTCATGTCGTTCA | cDNA Reverse primer #4 |
| SecA_cDNA_F5 | GGGACCGACACGTGATGAAG | cDNA Forward primer #5 |
| SecA_cDNA_R5 | AACCTGGAGCTTGCTTCTCC | cDNA Reverse primer #5 |
| SecA_cDNA_F6 | CCAGCGGGATCGGGTATATG | cDNA Forward primer #6 |
| SecA_cDNA_R6 | CCCACACTTCAGCCATCGTA | cDNA Reverse primer #6 |
| Primers for Intron 1 |  |  |
| SecA_Int_1_F1 | CTGGCTAAAACTGTCGCCCT | Forward primer#1, located in Intron 1 |
| SecA_Int_1_R1 | TTCCGTTCCCTGTATGCCAC | Reverse primer#1, located in Intron 1 |
| SecA_Int_1_F2 | TCTAACAGTATCCAGCCCCG | Forward primer#2, located in Intron 1 |
| SecA_Int_1_R2 | GGTTTCATGGCTGGGAGGTC | Reverse primer#2, located in Intron 1 |
| SecA_Int_1_F3 | TGGCATACAGGGAACGGAATC | Forward primer#3, located in Intron 1 |
| SecA_Int_1_R3 | TTGTGCTAGCCTACGACGTG | Reverse primer#3, located in Intron 1 |
| SecA_Int_1_F4 | AAGCGGAAGCCAGAACCAC | Forward primer#4, located in Intron 1 |
| SecA_Int_1_R4 | TTTTCGTTGGGCGTAACCCT | Reverse primer#4, located in Intron 1 |
| SecA_Int_1_F5 | CCGGAGCTTACCTGTCATTG | Forward primer#5, located in Intron 1 |
| SecA_Int_1_R5 | GTGTAGCACATAACTCCGCCT | Reverse primer#5, located in Intron 1 |
| SecA_Int_1_F6 | CCCTTGCCTATTTGATGCCAC | Forward primer#6, located in Intron 1 |
| SecA_Int_1_R6 | AAAGTTGGGAGCGCAGGTAG | Reverse primer#6, located in Intron 1 |
| SecA_Int_1_F7 | GATTATCCCATGAGCACCGC | Forward primer#7, located in Intron 1 |
| SecA_Int_1_R6 | ATCAGGGTTGAGTGTGCGAG | Reverse primer#7, located in Intron 1 |
| SecA_Int_1_F8 | AACCCAAGGTTCCAGTTCCA | Forward primer#8, located in Intron 1 |
| SecA_Int_1_R8 | TGCCTTCCAAGCAGATTTGAC | Reverse primer#8, located in Intron 1 |
| SecA_Int_1_F9 | TCGAACACGAGGACCCAATC | Forward primer#9, located in Intron 1 |
| SecA_Int_1_R9 | CTGAATTTCCCAGCGGCAAG | Reverse primer#9, located in Intron 1 |
| SecA_Int_1_F10 | ATAGCAGTCCAGCAGCAACT | Forward primer#10, located in Intron 1 |
| SecA_Int_1_R10 | GATCTAGTCACAAGCGGCGA | Reverse primer#10, located in Intron 1 |
| SecA_Int_1_F11 | ACAGCGACTCGTGCTTGAT | Forward primer#11, located in Intron 1 |
| SecA_Int_1_R11 | ACGGCCACGAACAAACAACT | Reverse primer#11, located in Intron 1 |
| SecA_Int_1_F12 | CTCTCCGCACTACTGGTTG | Forward primer#12, located in Intron 1 |
| SecA_Int_1_R12 | TGAAAATGTTTCAAGTCGTCGCA | Reverse primer#12, located in Intron 1 |
| SecA_Int_1_F13 | TCATTGGCCCTGATCCTTTCT | Forward primer#13, located in Intron 1 |
| SecA_Int_1_R13 | ACTACTTTGCAGTTTTCAGACAAT | Reverse primer#13, located in Intron 1 |
| Primers for *xan-m.48* breakpoint |  |  |
| 5kb_UP_xan-m.48_F | CCGCGACGGTAATTGGAGTA | *xan-m.48*-specific forward primer#1 |
| 5kb_UP_xan-m.48_R | GATGTCCCGAACGCGTCTAA | *xan-m.48*-specific reverse primer#1 |
| 1kb_UP_xan-m.48_F | TGCCGAAAACATCAGGAGACT | *xan-m.48*-specific forward primer#2 |
| 1kb_UP_xan-m.48_R | AAAGATTGTCTGCCTGCCCA | *xan-m.48*-specific reverse primer#2 |
| xan-m.48_bp_F | GTTAACAAGCTGGAGCCCGA | *xan-m.48*-specific forward primer#3 |
| xan-m.48_bp_R | GGTGAACGCAGCGGAATTAT | *xan-m.48*-specific reverse primer#3 |
| Primers for Intron 13 |  |  |
| SecA_Int13_F01 | TGGGGTTAACTTCGCAAAGAACAG | Forward primer#1, located in Intron 13 |
| SecA_Int13_F02 | CAGATAAGAGCAGGGTGTAGTGA | Forward primer#2, located in Intron 13 |
| SecA_Int13_F03 | ACTGCTCACTAAGACGTGCC | Forward primer#3, located in Intron 13 |
| SecA_Int13_F04 | ATCGCAAACTGCCATTGATGA | Forward primer#4, located in Intron 13 |
| SecA_Int13_F05 | AGAGAGTCCCTTGCTCAGGA | Forward primer#5, located in Intron 13 |
| SecA_Int13_F06 | ACACGACAACCAGTCAGCAA | Forward primer#6, located in Intron 13 |
| SecA_Int13_F07 | CTCATACCGCTGACGAAGGC | Forward primer#7, located in Intron 13 |
| SecA_Int13_F08 | ATTGGATCCGAGGCAGAGAC | Forward primer#8, located in Intron 13 |
| SecA_Int13_F09 | TGTCATTTCGCATTTCCCACC | Forward primer#9, located in Intron 13 |
| SecA_Int13_before_bp_F | ACTTGTCATTCTACACTTCCGATG | Forward primer, located in Intron 13 before the *xan-m.72* mutation |
| SecA_Int13_after_bp_F | CTTGCAGCCTTCCGCAAATC | Forward primer, located in Intron 13 after the *xan-m.72* mutation |
| SecA_Int13_after_bp_R | GATTTGCGGAAGGCTGCAAG | Reverse primer, located in Intron 13 after the *xan-m.72* mutation |
| Primers for intron 14 |  |  |
| SecA_Int14_F1 | TGTCATTTCGCATTTCCCACC | Forward primer#1, located in Intron 14 |
| SecA_Int14_R1 | CTGACGTCTCCGGTGCAATC | Reverse primer#1, located in Intron 14 |
| SecA_Int14_F2 | CTAGAGCAGCAGGTGGAGC | Forward primer#2, located in Intron 14 |
| SecA_Int14_R2 | TCGACGATCTTCAAGGTGGC | Reverse primer#2, located in Intron 14 |
| SecA_Int14_F3 | GCAGGTTGGACTACCGTGT | Forward primer#3, located in Intron 14 |
| SecA_Int14_R3 | AGAGGCAGGGAAGGTTTGAC | Reverse primer#3, located in Intron 14 |
| SecA_Int14_F4 | GTGGTCGCAAGAGACGCC | Forward primer#4, located in Intron 14 |
| SecA_Int14_R4 | TGATCGACACGTTCGCAGGA | Reverse primer#4, located in Intron 14 |
| SecA_Int14_F5 | GATCCCTGCACAGATGGCGA | Forward primer#5, located in Intron 14 |
| SecA_Int14_R5 | ATCGACCAGCGCTGCTATC | Reverse primer#5, located in Intron 14 |
| SecA_Int14_F6 | GGGGCCTATCTGCATCTTCTT | Forward primer#6, located in Intron 14 |
| SecA_Int14_R6 | GTAGGAAGGCGATCTTTGCC | Reverse primer#6, located in Intron 14 |
| SecA_Int14_F7 | GGTGCTACTCCTGTCGAACAT | Forward primer#7, located in Intron 14 |
| SecA_Int14_R7 | ATGTGCTGGAATGGATCGCC | Reverse primer#7, located in Intron 14 |
| SecA_Int14_F8 | GTCCTGTGCACCTCCGATTG | Forward primer#8, located in Intron 14 |
| SecA_Int14_R8 | GCAAGCGCGCAGAATAAGTT | Reverse primer#8, located in Intron 14 |
| SecA_Int14_F9 | CGGCCTTCGTACACTTCGAT | Forward primer#9, located in Intron 14 |
| SecA_Int14_R9 | AATCCTCGGCAATGCCATATC | Reverse primer#9, located in Intron 14 |
| SecA_Int14_F10 | ATTCGATGTCCTGGCCCTTC | Forward primer#10, located in Intron 14 |
| SecA_Int14_R10 | CGGTGCGTTCTAGGTCTGG | Reverse primer#10, located in Intron 14 |
| SecA_Int14_F11 | TAGACAAGGTCAGGGGCCAA | Forward primer#11, located in Intron 14 |
| SecA_Int14_R11 | AAGAGGCGTTGGGGATGAAT | Reverse primer#11, located in Intron 14 |
| SecA_Int14_F12 | TCCCCATGCGGAGAAGAAAAG | Forward primer#12, located in Intron 14 |
| SecA_Int14_R12 | ACAGAACCTGTCCCCTCCTA | Reverse primer#12, located in Intron 14 |
| Primers for Intron 15 |  |  |
| SecA_Int15_F1 | GCCTGGAGTCACTTATTGTGGA | Forward primer#1, located in Intron 15 |
| SecA_Int15_R1 | GCTGCTATGCATGATCGGGT | Reverse primer#1, located in Intron 15 |
| SecA_Int15_F2 | GCCATAGGCCGGTGGTA | Forward primer#2, located in Intron 15 |
| SecA_Int15_R2 | ATTCACCTTCCTCGTGGTCG | Reverse primer#2, located in Intron 15 |
| SecA_Int15_F3 | ACCCCACTAAAGCCTCCGAT | Forward primer#3, located in Intron 15 |
| SecA_Int15_R3 | AAACTGGTTAAGGCCCTCCC | Reverse primer#3, located in Intron 15 |
| SecA_Int15_F4 | TTTGCCCCTAATGGATGGCA | Forward primer#4, located in Intron 15 |
| SecA_Int15_R4 | CACCCGCTTCTAGCCCATAC | Reverse primer#4, located in Intron 15 |
| SecA_Int15_R01 | TCTGGGCCATCCTACTGTGT | Forward primer#5, located in Intron 15 |
| SecA_Int15_R02 | AGGATCTCGCTAGTGCTGGA | Reverse primer#5, located in Intron 15 |
| SecA_Int15_R03 | TCTGCTGAAGCTTAGTCGCC | Forward primer#6, located in Intron 15 |
| SecA_Int15_R04 | TCACCAACAGCTACTCTGCC | Reverse primer#6, located in Intron 15 |
| SecA_Int15_R05 | ATCCGGATGAGTGACAACTG | Forward primer#1, located in Intron 15 |
| SecA_Int15_R06 | TTAAGTTAGGAAGCGGGCGG | Reverse primer#1, located in Intron 15 |
| Primers for *xan-m.72* breakpoint |  |  |
| xan-m.72_1st_bp_F | CCGATATGGAAAAGAACATCCGA | Forward primer, specific for *xan-m.72* mutation breakpoint |
| xan-m.72_1st_bp_R | TCGGATGTTCTTTTCCATATCGG | Reverse primer, specific for *xan-m.72* mutation breakpoint |
| Primers for *xan-m.73* site-directed mutagenesis |  |  |
| SDM_xan-m.73_F1 | ATCAGCTACCAGATCTTCTTTCTGCAATTCCCGAAACTGTGCGGTATGAC | Forward primer, specific for *xan-m.73* mutation |
| SDM_xan-m.73_R1 | AATTGCAGAAAGAAGATCTGGTAGCTGATGCTCGCCAGGGTAATGGTTTC | Reverse primer, specific for *xan-m.73* mutation |
